# Autopsy case report of rapidly progressive iliopsoas abscess caused by *Escherichia coli* and *Prevotella bivia*

**DOI:** 10.1016/j.radcr.2026.02.004

**Published:** 2026-03-09

**Authors:** Koichi Saiki, Haruka Ikegami, Takuro Fujita, Tomoko Yokoyama, Yoshihiro Toyama, Masashi Ishikawa, Masahito Yamanaka

**Affiliations:** a Takamatsu Red Cross Hospital Department of Nephrology, Takamatsu-shi, Kagawa, Japan; b Takamatsu Red Cross Hospital Department of Radiology, Takamatsu-shi, Kagawa, Japan; c Takamatsu Red Cross Hospital Department of Pathology, Takamatsu-shi, Kagawa, Japan; d Takamatsu Red Cross Hospital Department of Urology, Takamatsu-shi, Kagawa, Japan

**Keywords:** Iliopsoas abscess, *Prevotella bivia*, Extrapleural abscess, Autopsy

## Abstract

Computed tomography-guided drainage or surgical drainage should be considered for psoas abscesses. Active surgical intervention is considered for gas-forming psoas abscesses; however, computed tomography-guided drainage may be selected, depending on the circumstances. In this case, Computed tomography was used to confirm the spread of the infection, but the autopsy revealed abscess formation outside the right pleural cavity, which was difficult to detect on computed tomography. *Prevotella bivia–*associated psoas abscesses are rare but have been previously reported.

A 48-year-old male had been undergoing hemodialysis for 5 years for diabetic nephropathy. Contrast-enhanced computed tomography revealed large right-sided predominant abscesses involving the iliopsoas muscle, an abscess with pneumoperitoneum in the para-aortic region, increased density in the perirenal adipose tissue, additional abscess formation, and right empyema. Blood cultures grew *Escherichia coli*, whereas drainage fluid cultures produced *Escherichia coli* and *Prevotella bivia*.

Despite treatment, improvement was minimal, and cardiac arrest occurred on the fourth day after transfer to our hospital.

## Introduction

The iliopsoas space is an extraperitoneal compartment that comprises the psoas major, psoas minor, and iliacus muscles. The psoas major muscle lies in close proximity to several organs including the sigmoid colon, appendix, jejunum, ureter, abdominal aorta, kidneys, pancreas, spine, and iliac lymph nodes. Therefore, infections of these structures can spread to the iliopsoas muscle. The rich blood supply in this region facilitates hematogenous dissemination from distant infection sites [1,2]. Symptoms of an iliopsoas abscess include fever, flank pain, back pain, abdominal pain, thigh mass, altered consciousness, and shock. However, diagnosis is often delayed because of nonspecific clinical presentation [3]. Blood cultures are positive in approximately 31.5% of the cases [4]. Advanced age, white blood cell count, platelet count, blood urea nitrogen, creatinine, and potassium levels have been shown to correlate with mortality [3]. Patients undergoing dialysis are at greater risk of developing bacterial infections than those without chronic kidney disease [5]. The reported mortality rates range from 5% to 15% [6]. In the present case, the patient complained of low back pain for 1 month; however, the condition was initially misattributed to an orthopedic cause. Following diagnosis of an iliopsoas abscess, the disease progressed rapidly leading to the formation of multiple abscesses throughout the body and ultimately resulting in death.

Computed tomography (CT)-guided drainage or surgical drainage should be considered for psoas abscesses. Active surgical intervention is considered for gas-forming psoas abscesses [3]; however, CT-guided drainage may be selected depending on the circumstances. CT scans are necessary to confirm the spread of the infection and perform drainage treatment [7], but confirmation may sometimes be difficult using CT. In the present case, autopsy confirmed the formation of an abscess outside the right thoracic cavity, which was difficult to identify on CT. *Prevotella bivia* (*P. bivia*)-associated psoas abscesses are rare but have been previously reported.

## Case Report

**Fig. 1:**
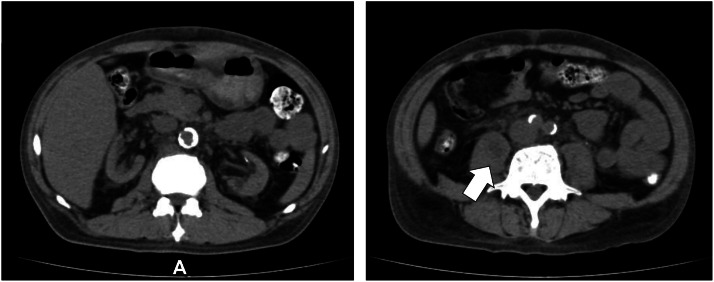

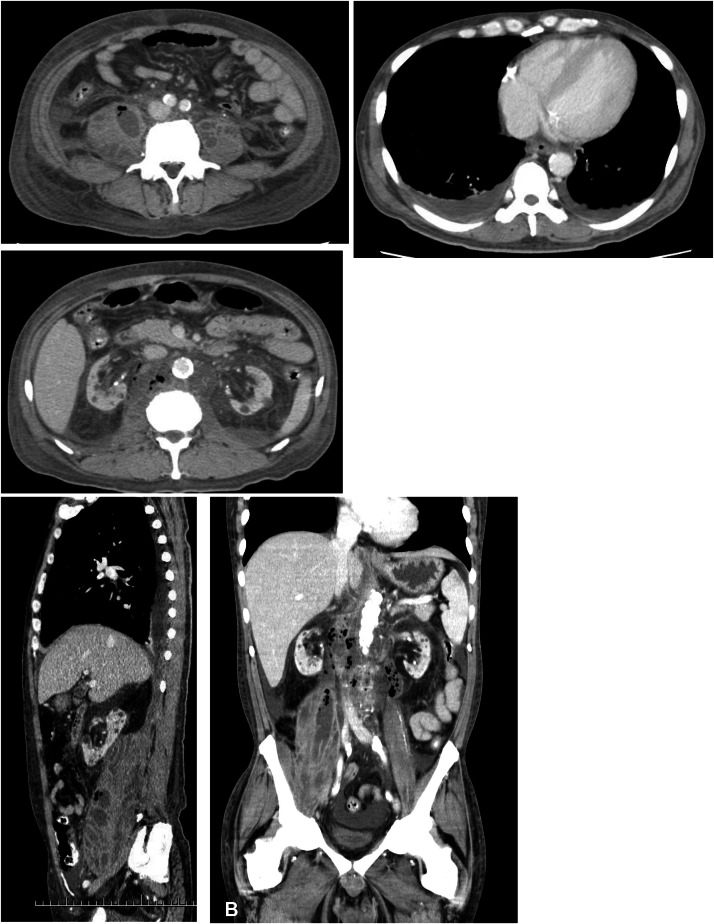
**(A)** Plain CT scan from 3 d prior shows edematous changes in the retroperitoneum and increased density of the perirenal adipose tissue. A 3-cm abscess is identified within the right psoas muscle (arrow). **(B)** Contrast-enhanced CT scan at admission shows severe right-sided predominated abscesses in the iliopsoas muscles, an abscess with pneumoperitoneum in the para-aortic region, further increased density of the perirenal fat, additional abscess formation, and right empyema. No intestinal perforation is observed.

A 48-year-old male had been undergoing hemodialysis for 5 years with diabetic nephropathy as the underlying disease. For approximately 1 month, he complained of discomfort in the right lower back and difficulty walking. The patient experienced hypotension during dialysis and reduced mobility, prompting a plain CT scan at the previous hospital 3 days earlier. Based on the imaging findings, an iliopsoas abscess was suspected, and the patient was transferred to our hospital via emergency transport. Physical examination revealed severe right lower back pain. The psoas sign was positive, and the patient had difficulty maintaining a supine position with legs extended. The plain CT scan obtained by the referring physician showed a 3-cm abscess in the left iliopsoas muscle accompanied by edematous changes in the retroperitoneum and increased perirenal fat density. Urinary tract infection was suspected as the source of infection. Contrast-enhanced CT performed upon admission revealed large right-sided predominant abscesses involving the iliopsoas muscles, an abscess with pneumoperitoneum in the para-aortic region, increased perirenal adipose tissue density, additional abscess formation, and right empyema. Bilateral pleural effusion was also observed. The imaging findings are shown in Fig. 1. Blood tests revealed markedly elevated levels of inflammatory markers, coagulation abnormalities, and increased lactate levels (Table 1).

**Table 1:** Laboratory examination on admission. A marked elevation in inflammatory markers was observed.

Blood chemistry			Hematology		
CRP	24.9	mg/dL	White blood cells	15950	/µL
Total protein	5.1	g/dL	Neutrophilis	92.4	%
Albumin	1.5	g/dL	Red blood cells	2.73	10^4^/µL
BUN	62.5	mg/dL	Hemoglobin	7.8	g/dL
Creatinine	6.27	mg/dL	Hematocrit	23.5	%
Uric acid	9.0	mg/dL	Platelets	14.0	10^4^/µL
Sodium	130	mEq/L			
Potassium	3.8	mEq/L	Coagulation		
Chloride	93	mEq/L	PT-INR	1.26	
Total bilirubin	1.4	mg/dL	APTT	30.5	seconds
AST	27	U/L	Fibrinogen	463	mg/dL
ALT	9	U/L	D-dimer	29.9	µg/ml
Arterial blood gas analysis (Room air)					
pH	7.402				
PaCO2	29.6	mmHg			
PaO2	71.5	mmHg			
Bicarbonate	19.4	mmol/L			
Lactate	2.75	mmol/L			

ALT, Alanine aminotransferase; APTT, Activated partial thromboplastin time; AST, Aspartate aminotransferase; BUN, Blood urea nitrogen; CRP, C-reactive protein; PaCO2, partial pressure of arterial carbon dioxide; PaO2, partial pressure of arterial oxygen; PT-INR, Prothrombin time-international normalized ratio.

**Fig. 2:**
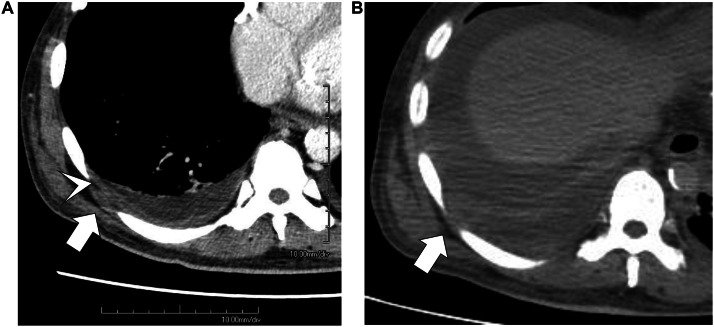
Right pleural space on contrast-enhanced CT at admission (A) and plain CT at death (B). Empyema with septal structures is observed in the right lung. The right intercostal muscles are partially displaced laterally (arrows), reflecting the formation of an extrapleural abscess. On contrast-enhanced CT, extrapleural fat tissue appears hypodense but no proliferation is noted (arrow head). Its structure also appears indistinct.

**Fig. 3:**
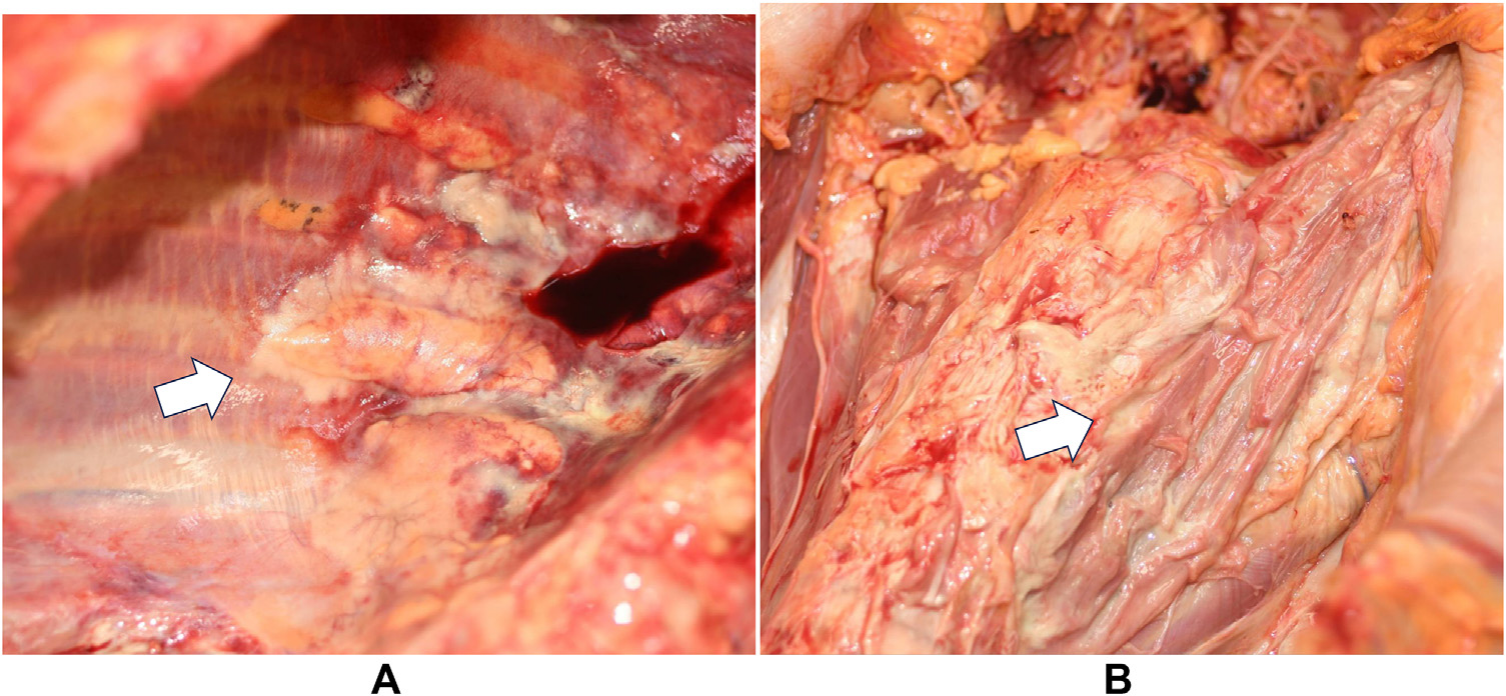
Autopsy photographs: **(A)** Right thoracic cavity viewed from the caudal side. Right psoas muscle internally replaced by abscess tissue (arrow). **(B)** Photograph from the head side. Abscess formation is observed in the right extrapleural space (arrow).

Percutaneous drainage was performed on both iliopsoas muscles, drainage tubes were placed, and treatment was initiated with meropenem and vancomycin. Blood cultures grew *Escherichia coli* (*E. coli*), whereas drainage fluid cultures yielded *E. coli* and *P. bivia*. The *E. coli* strain was an extended-spectrum β-lactamase-producing bacterium, but it remained highly susceptible to the administered antibiotics. CT-guided puncture of both iliopsoas muscles was completed without complications, and drainage yielded 10 and 25 mL from the left and right sides, respectively. On the following day, 11 mL was drained from the left side and 3 mL from the right side, whereas on the third day, 19 mL and 18 mL were drained from the left and right sides, respectively. Upon arrival at our hospital, the patient’s blood pressure was 122/61 mmHg, pulse rate was 80 bpm, and SpO_2_ was 95% (on room air), indicating a stable condition. However, despite fluid resuscitation on the following day and thereafter, the systolic blood pressure did not reach 100 mmHg, and scheduled dialysis could not be performed. Severe pain on both sides of the lower back and difficulty in lying supine persisted. On the fourth day after admission to our hospital, the patient experienced sudden severe abdominal pain, upward deviation of the eyes, and a decline in consciousness on the Japan Coma Scale 300, followed by cardiac arrest. Postmortem plain CT revealed increased bilateral empyema with septal structures (Fig. 2). The family declined resuscitation and death was confirmed on the same day. Autopsy was performed after obtaining informed consent from the patient’s family. Upon removal of the drainage tube before the autopsy, persistent purulent discharge was observed at the removal site. The autopsy revealed bilateral iliopsoas abscesses, extensive retroperitoneal and perirenal abscesses, and abscess formation in the right extrapleural space. The iliopsoas muscle was almost entirely replaced by the abscess tissue. A significant amount of residual abscess material remained, indicating that adequate drainage had not been achieved. Intestinal injury or myelitis was not observed. Histologically, the inflammation extended along the aorta into the right pleural cavity up to the seventh intercostal space. Inflammation and abscess formation were observed around the thoracic aorta and esophageal adventitia. Inflammation was observed in the kidneys, adrenal glands, pancreas, and periaortic adipose tissue. However, no direct infiltration into these organs was observed. The photographs are shown in Fig. 3.

## Discussion

*P. bivia* and *E. coli* were isolated from the abscess culture, and *E. coli* was detected in the blood culture. Both organisms are gram-negative rods (GNR). In non-gas-forming iliopsoas abscesses, the primary causative microorganisms are gram-positive cocci (GPC), whereas in gas-forming iliopsoas abscesses, the incidence of GPC and gram-negative bacilli are comparable [3]. Iliopsoas abscesses are classified as primary or secondary [1,3]. Primary iliopsoas abscesses accounted for approximately 21.8% of the cases, whereas secondary cases accounted for approximately 78.2%. The sources of infection for secondary iliopsoas abscesses included skeletal (39.5%), gastrointestinal (19.4%), and genitourinary (13.7%) origins. The most common skeletal source is spinal disc inflammation, whereas diverticulitis and tumors are prevalent in the gastrointestinal tract. Urinary tract infections and renal abscesses are common [4]. In this case, the infection may have disseminated from the kidneys. *P. bivia* is an anaerobic GNR that commonly resides in the urogenital tract but can also be isolated from the oral cavity [8]. *E. coli* is the most frequent causative organism of iliopsoas abscesses [4]. Although reports of iliopsoas abscesses caused by *Prevotella* spp. are rare, several cases have been described [9,10]. Gas-forming iliopsoas abscesses progress rapidly [3], and *P. bivia* may have contributed to the rapid deterioration observed in this case.

Differential diagnoses of psoas abscesses include hematoma, tumor, and tuberculous abscesses [11,12]. Abdominal tuberculosis abscesses arise from the ingestion of *Mycobacterium tuberculosis* in the digestive tract or through hematogenous spread from infected organs, primarily the lungs. Lymph node enlargement is commonly observed on CT, presenting as isolated masses or masses adhering to surrounding organs. On contrast-enhanced CT, these masses exhibited a central hypoattenuating core with peripherally hyperattenuating enhancement. In the early stages, they may form granulomas with homogeneous enhancement. Differential diagnosis of tumors can be difficult, and a biopsy may be necessary for final diagnosis. Metastatic lymph node enlargement due to lymphoma or tumors demonstrates uniform contrast enhancement on contrast-enhanced CT [11]. Reports of iliopsoas hematoma indicate that contrast-enhanced CT may reveal low-density areas within a high-density hematoma, and in some cases, contrast agent leakage may be observed, suggesting ongoing bleeding. Several bilateral cases have been previously reported [12].

Additionally, although rare, we observed the formation of an abscess in the right extrapleural space, complicating the retroperitoneal abscess. Based on the autopsy findings, inflammation spread from the area around the aorta into the thoracic cavity, and the inflammation was considered to be continuously extending from the retroperitoneum. The extrapleural space lies between the inner surface of the ribs and the parietal pleura and contains fatty tissue, loose connective tissue, lymph nodes, and blood vessels [13]. Reports showed that inflammation caused proliferation of poorly absorbed extrapleural adipose tissue. In cases of extrapleural hematoma, the extrapleural adipose tissue is displaced inward. When air accumulates extrapleurally, reticular linear septa are observed in the extrapleural space [13]. Cases of abscess formation accompanied by thickening and calcification of the parietal pleura and increased extrapleural adipose tissue have been reported in patients with chronic tuberculous empyema [13,14]. In this case, contrast-enhanced CT on admission and plain CT at autopsy showed that the right intercostal muscles were partially displaced laterally and structurally obscured, possibly reflecting abscess formation in the right extrapleural space. Extrapleural adipose tissue was identified as low attenuation, but no proliferation was observed. This was likely due to the rapid progression of inflammation. No wall thickening of the parietal pleura was observed, and the presence of purulent pleural effusion made it difficult to identify abscess formation on the CT images.

Treatment of iliopsoas abscesses typically involves a combination of antibiotic therapy and drainage. Surgical intervention, either open or CT-guided percutaneous drainage, is generally indicated when the abscess diameter exceeds 3 cm [15]. Currently, no clear criteria exist for determining whether to perform CT-guided or surgical drainage. However, surgical drainage reportedly achieved higher cure rates than CT-guided drainage for gas-forming iliopsoas abscesses [3]. In the present case, the patient’s vital signs were not sufficiently stable to tolerate surgery; therefore, CT-guided drainage was selected. In adult males, the iliopsoas muscle volume is approximately 400 mL per side [16]. The right iliopsoas muscle was almost entirely replaced by the abscess tissue, suggesting the presence of a large amount of purulent material. Although only a small volume of the fluid was drained, the high viscosity of the discharge was likely a contributing factor. Some reports have indicated that saline irrigation during drainage may help achieve more complete evacuation of purulent material [17]. In this case, the initial management focused on drainage of the iliopsoas abscess; however, the patient’s condition deteriorated. Drainage of the retroperitoneal abscess with gas formation should have been performed initially.

## Conclusion

We encountered a case of an extremely rapidly progressing gas-forming iliopsoas abscess that likely involved *P. bivia*. The condition progressed rapidly and required aggressive drainage therapy. Autopsy revealed massive purulent discharge extending from the psoas muscle, retroperitoneum, and perirenal space to the right extrapleural space. This case suggests that an abscess may have developed in the right extrapleural space in association with an iliopsoas abscess.

## Ethical approvals

All procedures involving human participants performed in this study were in accordance with the ethical standards of the institutional and national research committees, the Declaration of Helsinki of 1964, and its subsequent amendments or comparable ethical standards.

## Patient consent

Informed consent was obtained from individual participants in the study.
